# Editorial: Cognitive factors in bilingual language processing

**DOI:** 10.3389/fpsyg.2022.1055759

**Published:** 2022-10-13

**Authors:** Yan Jing Wu, Koji Miwa, Haoyun Zhang

**Affiliations:** ^1^Faculty of Foreign Languages, Ningbo University, Ningbo, China; ^2^Graduate School of Humanities, Nagoya University, Nagoya, Japan; ^3^Centre for Cognitive and Brain Sciences, University of Macau, Macau, Macau SAR, China

**Keywords:** bilingualism, cognition, psycholinguistic, neuroscience, translation

In this Research Topic, we received a wide range of submissions concerning several aspects of language processing in bilinguals. It is our greatest honor to review such a fascinating collection of articles, but space limits allowed publishing only a small fraction of them. Here, we are delighted to present eleven articles, ten original research based on empirical studies and one review. Their main findings and perspectives are summarized below.

At the center of bilingual language processing is the question of how bilinguals access (and control) lexical-semantic representations of the two languages. Experimental psychology and neuroscience have made the case that when bilingual individuals speak and read in one language, the other language is simultaneously activated, a phenomenon sometimes referred to as non-selective activation or cross-language interactions. Zeng et al. examine the effects of task demands and L2 proficiency on cross-language interactions by comparing the performance of Chinese-English speakers with high and low proficiency in English on a semantic and a lexical task. Their findings add to the existing literature of cross-language interactions and shed new light on classic psycholinguistic models.

A classic paradigm in the study of cross-language interactions involves the use of cognates, words that share the same or similar semantic contents and lexical (phonological and orthographic) forms between bilinguals' two languages. Typically, cognates are processed faster as compared to non-cognate controls by bilinguals (i.e., the cognate facilitation effect). However, Frances et al. show that Spanish-English cognates with orthographic similarities lead to greater response time and less accuracy, indicating an unexpected cognate inhibitory effect. Interestingly, the effect which is found in an auditory task is affected by the speaker's accent. The accent of the bilingual's native language (but foreign to that of the L2) reduces this inhibitory effect. While the exact mechanism is still under exploration, Frances et al.'s results bring new insights into mental operations underlying the cognate effect, an observation that is almost as old as research in bilingualism itself. Unlike cognates in alphabetic languages, those in two languages with different script systems have completely unrelated orthography while sharing semantics and phonology in common. Using event-related brain potentials, Chen et al. investigate how word concreteness affects the processing of cross-script cognates in a lexical decision task. A masked translation priming paradigm involving both forward and backward translation directions is applied to prevent spurious effects due to participants' awareness of the cognate status of the critical stimuli. Results of Chen et al. highlight an interaction between semantic (i.e., word concreteness) and lexical (i.e., phonology) processing of cross-script cognates.

Language acquisition context is another important factor that affects language processing in bilinguals and the interaction between L1 and L2. Using a lexical decision task, Hevia-Tuero et al. compare Spanish-English bilingual children from a monolingual school to those from a bilingual school with a focus on cross-language (phonological) interference. The lexical decision task involves real words and pseudohomophones in both Spanish and English. Hevia-Tuero et al.'s results show the effects of both instructional language (i.e., language acquisition context) and level of education on the ability to control L1-to-L2 interference on phonological access and also at the level of grapheme-phoneme correspondence regularities. Short-term language training is an effective way to examine language (L2) acquisition process in an experimental context. Deng et al. train a group of Chinese learners of English with subject-verb agreement in English and test the same group of participants with a different set of stimuli that involve the same syntactic structure. A syntactic transfer effect is found as the processing of grammatically incorrect sentences induces a larger P600 effect, classic ERP index of syntactic violation, as compared to correct sentences, suggesting that even for late L2 learners, syntactic knowledge can be developed with a relatively short period of training.

Embodiment is another perspective by which language processing is studied and compared between bilinguals and monolinguals. It has been shown that the sensorimotor system is involved (i.e., language embodiment) when advanced bilinguals process words in L2, but less is known regarding L2 beginners. Bai and He examine how less advanced bilinguals process spatially associated words in L2 and show that the degree of embodiment as indexed by automatic activation of sensorimotor response is dependent on the level of task demands.

Spoken word segmentation, a process in which listeners spontaneously “cut” continuous utterances into meaning parts during oral communication, presents a serious problem for less advanced L2 learners. Yang et al. study the cognitive mechanisms of spoken word segmentation by characterizing the interaction between spoken word segmentation efficiency on one hand and cognitive inhibition, cognitive flexibility, and L2 listening proficiency on the other hand. Yang et al.'s findings support an interactive model as they show that both the bottom-up and top-down processes determine spoken word segmentation performance in bilinguals. In a similar context, Guan et al. investigate to what extent bilingual listeners can take advantage of pitch accents as a memory cue when recalling contents of spoken discourse that was presented in L2. Pitch accents are detailed auditory information that can be used as a processing cue to facilitate speech comprehension and recalls. In Guan et al., both L2 proficiency and working memory are considered as cognitive factors in an auditory recognition task, where signal detection theory is applied in the analysis of the data.

Interpretation and translation are unique processing contexts that are often studied as an independent subject of bilingualism. Zhao takes a novel approach to interpretation by examining how L2 proficiency, working memory, and anxiety levels affect the fluency when interpreting speeches from L2 to L1, effectively taking considerations of linguistic, cognitive, and emotional factors in the same functioning context of bilinguals. In addition to its contribution to the growing literature of interpretation, the findings of Zhao have real-life implications for practitioners. Similar to Zhao, but in a visual translation context, Yuan and Tu study the affective valence of visual imagery expressions when English-Chinese bilinguals read a classic Chinese poem. While poetry comprehension in L2 is an underdeveloped subject in bilingualism research, Yuan and Tu's study reveals how translation strategies and cultural factors affect emotional responses to words that are intended, in the native language of the original poem, to stimulate visual imagination and emotional reactions associated with the imagination.

Knowledge of cross-language variances is the foundation of research in bilingualism. In the review article by Li, semantic fusion, which is the realization and integration of multiple semantic roles in one syntactic element, is investigated and compared between Chinese and English using corpus analysis. Since merging semantic roles is a critical step in event alignment during sentence comprehension, variances in the semantic fusion process between languages could be a potential factor affecting language processing in bilinguals. Interestingly, however, Li shows highly comparable patterns of corpus data when short sentences with two verbs designating a double semantic role (i.e., patient and agent) in one noun are analyzed between Chinese and English, suggesting universality in syntactic processing across languages.

## Author contributions

All authors listed have made a substantial, direct, and intellectual contribution to the work and approved it for publication.

## Funding

This work was funded by the National Social Science Fund of China (21AYY014).

## Conflict of interest

The authors declare that the research was conducted in the absence of any commercial or financial relationships that could be construed as a potential conflict of interest.

## Publisher's note

All claims expressed in this article are solely those of the authors and do not necessarily represent those of their affiliated organizations, or those of the publisher, the editors and the reviewers. Any product that may be evaluated in this article, or claim that may be made by its manufacturer, is not guaranteed or endorsed by the publisher.

